# Gene expression is differentially regulated in skeletal muscle and circulating immune cells in response to an acute bout of high-load strength exercise

**DOI:** 10.1186/s12263-017-0556-4

**Published:** 2017-03-03

**Authors:** Gyrd O. Gjevestad, Håvard Hamarsland, Truls Raastad, Inger Ottestad, Jacob J. Christensen, Kristin Eckardt, Christian A. Drevon, Anne S. Biong, Stine M. Ulven, Kirsten B. Holven

**Affiliations:** 1 Department of Nutrition, Institute of Basic Medical Sciences, Faculty of Medicine, University of Oslo, P.O. Box 1046, Blindern, 0317 Norway; 2 Centre for Research and Development, TINE SA, P.O. Box 7, Kalbakken, 0902 Oslo Norway; 3 Department of Physical Performance, Norwegian School of Sport Sciences, P.B. 4104 USA, 0806 Oslo, Norway; 4 The Lipid Clinic, Oslo University Hospital Rikshospitalet, P.O. Box 4950, Nydalen, 0424 Oslo Norway; 5 National Advisory Unit on Familial Hypercholesterolemia, Department of Endocrinology, Morbid Obesity and Preventive Medicine, Oslo University Hospital, P.O. Box 4950, Nydalen, 0424 Oslo Norway

**Keywords:** Peripheral blood mononuclear cells, Skeletal muscle, mRNA, Resistance exercise, Muscle regeneration, Cytokines

## Abstract

**Background:**

High-intensity exercise induces many metabolic responses. In is unknown whether the response in the peripheral blood mononuclear cells (PBMCs) reflects the response in skeletal muscle and whether mRNA expression after exercise can be modulated by nutritional intake.

The aims were to (i) investigate the effect of dairy proteins on acute responses to exercise in skeletal muscle and PBMCs measuring gene expression and (ii) compare this response in young and older subjects.

**Methods:**

We performed two separate studies in young (20–40 years) and older subjects (≥70 years). Subjects were randomly allocated to a milk group or a whey group. Supplements were provided immediately after a standardized exercise session. We measured mRNA expression of selected genes after a standardized breakfast and 60/120 min after finishing the exercise, using RT-qPCR.

**Results:**

We observed no significant differences in mRNA expression between the milk and the whey group; thus, we merged both groups for further analysis. The mRNA expression of *IL6*, *TNF*, and *CCL2* in skeletal muscle increased significantly after exercise compared with smaller or no increase, in mRNA expression in PBMCs in all participants. The mRNA expression of *IL1RN*, *IL8*, and *IL10* increased significantly in skeletal muscle and PBMCs. Some mRNA transcripts were differently regulated in older compared to younger participants in PBMCs.

**Conclusions:**

An acute bout of heavy-load strength exercise, followed by protein supplementation, caused overlapping, but also unique, responses in skeletal muscle and PBMCs, suggesting tissue-specific functions in response to exercise. However, no different effects of the different protein supplements were observed. Altered mRNA expressions in PBMCs of older participants may affect regenerative mechanisms.

**Electronic supplementary material:**

The online version of this article (doi:10.1186/s12263-017-0556-4) contains supplementary material, which is available to authorized users.

## Background

High-intensity physical exercise induces several metabolic responses and represents a major challenge to whole-body homeostasis. Numerous adaptations take place to meet this challenge, both locally (including changes in mRNA expression and protein levels) and systemically (including hormonal signaling and organ crosstalk) [1–3]. Ultimately, these events will promote altered expression of the key proteins in skeletal muscle [4–6], as well as the immune system [5, 7, 8]. To study alternations in gene expression levels of the immune system in response to short- and long-term nutritional interventions, peripheral blood mononuclear cells (PBMCs) have been used as a surrogate model [9, 10]. Whether PBMCs are a good model system for studying gene expression levels in skeletal muscle in response to exercise is less known.

A growing number of studies show that mRNA expression in the recovery phase from exercise can be modulated by nutritional intake [11–16]. Intake of sufficient energy and protein, in combination with regular exercise, may promote muscle protein synthesis [17–20]. Dairy proteins have been hypothesized to modulate inflammation by having anti-inflammatory properties [21–23]. However, we know little about how different dairy proteins affect mediators of the immune system after an acute bout of exercise.

Aging is associated with a range of cellular and biochemical changes, including increased inflammation, altered cell migration and cell signaling [24], and may be an important factor determining the molecular signature of an acute bout of exercise. Previous studies suggest an attenuated expression of markers released after exercise in old compared to young subjects, in skeletal muscle [25–32], in serum [33] as well as in the cells of the immune system [34].

The aims of the present study were to (i) investigate the effect of dairy proteins on acute responses to exercise in skeletal muscle and PBMCs measuring mRNA expression of selected genes and (ii) compare this response in young and older subjects.

## Methods

### Study populations and experimental design

We performed two separate acute exercise studies, where we supplemented young (20–45 years) and older (≥70 years) adults with dairy products based on regular milk or whey protein. Both studies were conducted at the Norwegian School of Sports Sciences. The first study (study 1) was conducted during the fall of 2013 and the second study (study 2) during the fall of 2014 and the spring of 2015. Written informed consent was obtained from all participants.

**Fig. 1:**
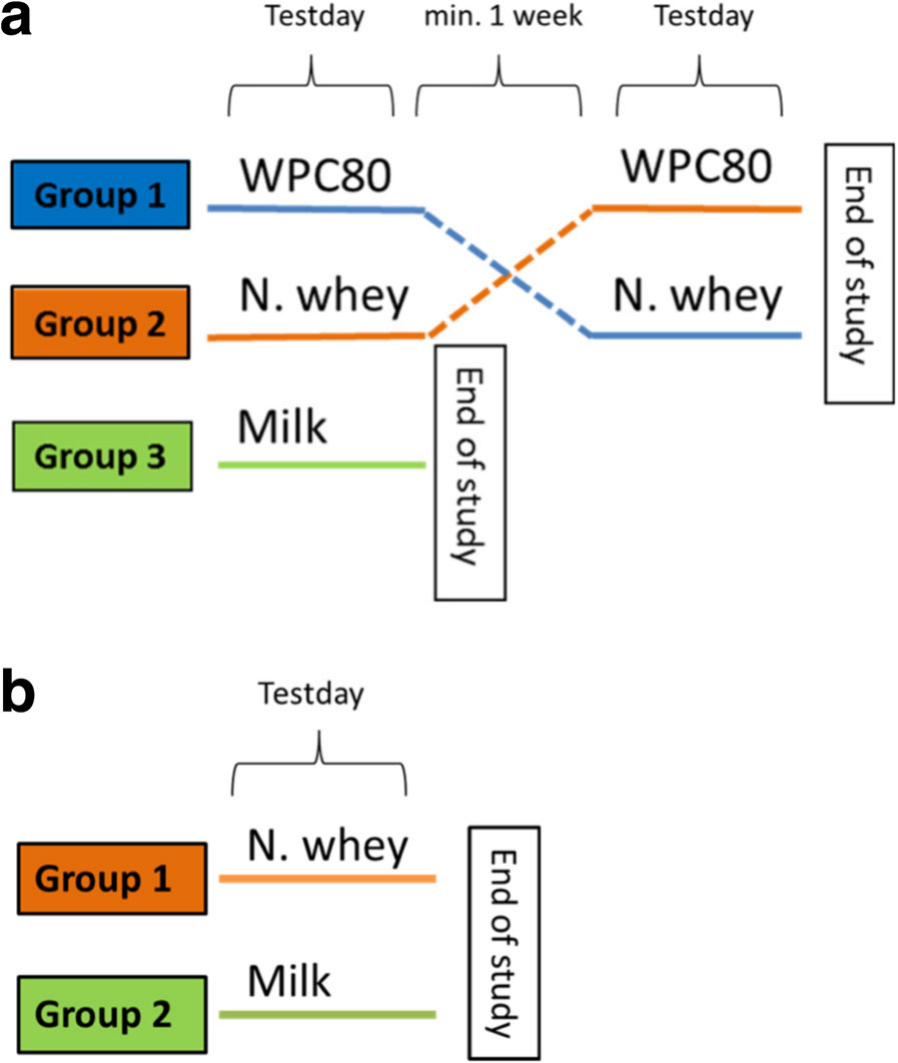
**a** In study 1, participants were randomized into one of two groups, receiving either milk or whey supplements. Participants in the whey group were testing two different whey products in a randomized order. All groups performed the exercise. There was a minimum 1 week between test days in the crossover part of the study. **b** In study 2, participants were randomized into one of two groups, receiving either milk or native whey supplements in combination with exercise. *WPC80* whey protein concentrate with 80% protein, *N whey* native whey

In study 1, 24 resistance-trained young (mean age; 25.0 ± 3.5 years) and 17 recreationally active older (mean age; 74.2 ± 3.8) healthy men and women were randomly allocated into a milk group or a whey group. Further, subjects in the whey group were randomized to receive whey protein concentrate (WPC80) or native whey on the first test day in a crossover design (Fig. 1a). Information about the training habits in the last 6 months before inclusion were obtained. In study 2, 25 young (mean age; 28.9 ± 5.8 years) and 24 older (mean age; 73.7 ± 3.4 years) healthy, untrained men and women, were randomized into a milk group or a native whey group (Fig. 1b). In both studies, the appropriate test drink was consumed immediately after performing a standardized exercise session. Participants reported to be non-smokers with no cardiovascular diseases or diabetes. In study 1, three older subjects had prescribed medication for high blood pressure and two took statins. In study 2, one older subject had prescribed anticoagulants and six took statins. One older participant and two young subjects did not complete the study and were excluded from further analysis in study 1, whereas one young participant did not complete the study and was excluded from further analysis in study 2.

On the morning of each test day, subjects reported to the laboratory in a fasted state. Upon arrival, they were served a standardized breakfast consisting of oatmeal, water, rapeseed oil, and sugar (50 energy percent (E%) from carbohydrate, 8 E% from protein and 42 E% from fat). All subjects finished the breakfast within 20 min. One day before the exercise, and until the last performance test was completed the following day, all participants followed a standardized diet.

### Protein supplements

The supplements were based on regular milk or whey protein (WPC80 or native whey proteins). The test products were isocaloric and contained 20 g of protein (27 E%), 39 g carbohydrates (52 E%), and 7 g fat (21 E%), providing approximately 300 kcal per serving. Thus, the main difference between test products was the amino acid composition, as illustrated in Table 1. Further, the production method for WPC80 differed from that of native whey as native whey was produced at low temperatures (below 60 °C). In both studies, the supplements were provided in identical packages to ensure blinding of both the providers and the participants, although the products were labeled with color codes to ensure that the participants received the correct products. The color-coding was provided by the producer and was not revealed until the interventions and statistical analyses were completed. All products had the same flavor, color, and appearance.

**Table 1:** The main difference between test products

Amino acids (% of total)	Low-fat milk	WPC80	Native whey^a^
Alanine	3.2	4.8	4.6
Arginine	3.3	2.4	2.7
Aspartate	7.5	10.6	10.8
Cysteine	0.8	2.1	2.5
Phenylalanine	4.7	3.3	3.8
Glutamic acid	20.4	17.1	17.4
Glycine	1.9	1.9	1.9
Histidine	2.7	1.9	2.2
Isoleucine	4.9	6.0	5.3
Leucine	9.6	10.3	11.8
Lysine	8.2	9.2	9.9
Methionine	2.5	2.1	2.3
Proline	9.7	6.4	5.5
Serine	5.6	5.4	4.7
Threonine	4.4	7.0	4.9
Tyrosine	3.5	2.1	2.9
Valine	6.0	5.7	5.3
Tryptophan	1.3	1.7	2.0
EAA	41.4	45.3	45.2

^a^Mean values for native whey from studies 1 and 2

### Exercise protocols

**Fig. 2:**
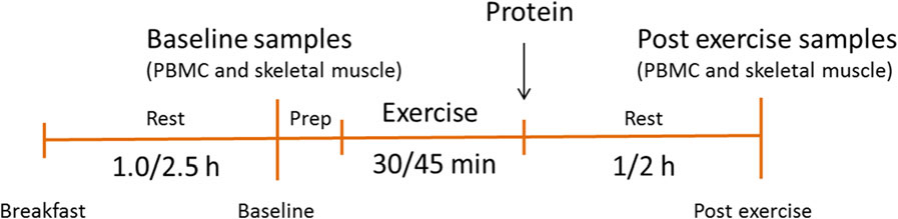
Timeline of study design and times of sampling in study 1 and study 2. A standardized breakfast was served upon arrival, 2.5 h (study 1) or 1 h (study 2) before the baseline samples were drawn. Thirty minutes after baseline, a 30 (study 1)- or 45 (study 2)-min exercise session was performed directly followed by intake of a protein drink. Post exercise samples were drawn 1 (study 1) or 2 (study 2) h after finishing the drink

In study 1, the young participants had experience with strength training prior to inclusion, whereas the older subjects had been recreationally active. To become accustomed with the exercise session, young participants performed the exercise session twice prior to the test day, whereas older subjects performed the exercise session until they were familiar with the exercise (average 4.4 times, maximum of 6 times). These exercise sessions were also used to determine the workload needed for each participant. On the test day, the exercise session lasted for 30 min and included 4 ×8 repetition maximum (RM) of leg press and knee extension, with a new set starting every third minute. Baseline was defined as 2.5 h after the standardized breakfast was served. The exercise session started approximately 30 min after baseline and was immediately followed by intake of a test drink (3.5 h after breakfast was served). Subjects had to finish the test drink within 5 min. Blood samples and skeletal muscle biopsies were collected at baseline and 1 h after completing the exercise (Fig. 2).

Participants in study 2 were untrained, but they were made familiar to the exercise session and the 10 RM training loads were determined. On the test day, the exercise session lasted for 45 min and included 3 × 10 RM of hammer squat, leg press, knee extension, bench press (chest press in the older subjects), seated rowing, and 1 × 10 RM and 2 × 10 RM in a close grip pull-down and shoulder press, respectively. A new set started every third minute. In this study, baseline was defined as 1 h after breakfast was served. The exercise session started approximately 30 min after baseline and was immediately followed by the intake of a test drink (2.15 h after breakfast was served), which had to be finished within 5 min. The blood samples and skeletal muscle biopsies were collected at baseline and 2 h after completing the exercise (Fig. 2).

### Sampling and sample preparation

The venous blood samples were collected in BD Vacutainer® CPT^TM^ cell preparation tubes with sodium heparin (Becton Dickinson, NJ, USA) and in BD Vacutainer® SST^TM^II Advanced tubes for serum (Becton Dickinson, NJ, USA). Within 2 h of blood collection, PBMCs were collected by density gradient centrifugation (1636*g*) for 25 min at room temperature. The cells were washed twice (300*g*, 10 min) in PBS without CaCl_2_ and MgCl_2_. After the last washing step, excess PBS was discarded. The pellet was dissolved in the remaining liquid and transferred to an Eppendorf tube, centrifuged (13000*g*, 3 min, 4 °C) and frozen at −80 °C until further analysis. The serum samples were left on the bench top for at least 30 min to ensure complete coagulation, centrifuged (1550*g*, 15 min at room temperature) and frozen at −80 °C until further analysis.

The muscle biopsies from the *m. vastus lateralis* were collected at the same time points as the blood samples with a modified Bergstrom technique [35]. The biopsies were immediately cleaned from the blood and connective tissue in physiological salt water at 4 °C, immersed into RNAlater® solution (Ambion, Texas, USA), and stored overnight at 4 °C. The following day, the biopsies were transferred and stored at −80 °C until further analysis. The biopsies were taken from the left leg, and the same incision was used for both biopsies, but the needle was placed in an angle so that the two sample sites were separated by at least 5 cm. The second sample was always collected proximal to the first sample.

### Isolation of mRNA

mRNA was isolated from thawed PBMCs using Qiagen RNease Mini Kit in accordance with the protocol provided (QIAGEN GmbH, Germany). In brief, PBMC pellets were lysed and homogenized in the presence of a highly denaturing guanidine thiocyanate-containing buffer. Ethanol was added to provide appropriate binding conditions before the samples were applied to an RNeasy Mini spin column. Contaminants were washed out using buffers in the kit. A one-column DNase digest (QIAGEN GmbH, Germany) was used to remove potential DNA contaminants. High-quality RNA was eluted in 30 μL of RNase free water and frozen at −80 °C until further analysis. In study 2, this protocol was conducted using the QiaCube (QIAGEN GmbH, Germany) in accordance with the protocol RNeasy Mini Kit with Qiashredder columns and DNase digest. High-quality RNA was eluted in 30 μL RNAse free water and frozen at −80 °C until further analysis.

Without thawing, the muscle biopsies were ruptured using a mortar and pestle (study 1) or a CryoGrinder (study 2), followed by homogenization in Qiazol (QIAGEN GmbH, Germany). Chloroform (1:5, *v*:*v* for chloroform:Qiazol) was added and the samples centrifuged (12000*g*, 15 min, 4 °C). The upper phase, with mRNA, was transferred to a fresh tube before adding ethanol. The samples were applied to a miRNeasy column using the protocol provided by QIAGEN GmbH (Germany). The protocol was performed manually in study 1, whereas the QiaCube from QIAGEN GmbH (Germany) was used in study 2. Thirty-microliter high-quality RNA was eluted in RNase free water and frozen at −80 °C until further analysis.

RNA quantity was measured using NanoDrop-1000 (NanoDrop Technologies, Inc., Delaware, USA), and RNA quality was checked with Agilent 2100 Bioanalyzer (Agilent Technologies, Inc., California, USA). All samples included in further analyses had a RIN value above 6. One young participant from study 1 and two subjects (one young and one older) from study 2 were excluded from further analysis, due to missing PBMC samples. Five participants (one young and four older) and 19 participants (15 young and six older) from study 1 and 2, respectively, were excluded from further analysis due to low RNA quality isolated from the muscle samples.

### Synthesis of cDNA

Complementary DNA (500 ng) was made using a RNA to cDNA kit from Applied Biosystems (Applied Biosystems, UK) in accordance with manufacturer’s protocol. The samples were stored at −20 °C for further analysis.

### RNA analysis by RT-qPCR

We monitored the mRNA expression levels of 24 mRNA transcripts, using TaqMan Low-Density array (TLDA) cards from Applied Biosystems (UK). Eighteen of the transcripts were analyzed in both studies (Additional file 1; overview of mRNA transcripts). mRNA transcripts were selected based on a thorough literature search investigating the effect of acute exercise on gene expression levels in PBMCs [36] as well as skeletal muscle [37]. Moreover, the selection was based on studies where effects of dairy products on markers of inflammation were investigated [38]. TLDA cards were used on a 7900 HT Applied Biosystems RT-qPCR machine (Applied Biosystems, UK). The Ct values were analyzed using SDS 2.4 (Applied Biosystems, UK), and further transferred to ExpressionSuite Sofware v1.0.3 (Applied Biosystems, UK). We normalized the Ct values to TATA box-binding protein (TBP) mRNA transcripts. Fold changes in mRNA transcripts from baseline to after the exercise session were calculated by dividing 2^−ΔCtpost exercise^ with 2^−ΔCtbaseline^, using the 2^−ΔΔCt^-method [39].

### Cytokine measurements

The serum level of IL6 was determined using a high-sensitivity Quantikine HS ELISA Kit (R&D Systems Inc., Minneapolis, USA), according to the protocol. All samples were measured in duplicates.

## Statistics

Power calculations were only made for the primary outcome of the study, but the number of participants included in the mRNA expression analysis reported here is in line with the number included in similar studies exploring potential changes of exercise on mRNA expression levels [40–44].

First, we examined changes in mRNA expression and serum levels from the acute bout of exercise between drinks, as well as between young and older subjects, in both studies separately. Since we found no differences in gene expression and serum levels between WPC80 and native whey in study 1, we decided to combine the results from the whey groups when comparing the whey group with the milk group. After inspecting the results of study 1 and study 2 individually, we also found these to be similar; thus, we decided to combine the results from study 1 and study 2 into one data set.

All data were checked for normality. For non-parametric data, we used Wilcoxon signed-rank test for repeated, paired measurements, and the Mann-Whitney test for independent measurements. Fold changes (relative quantification) were calculated using the ratio of 2^−ΔCt after exercise^ to 2^−ΔCt baseline^ [39]. For parametric data, differences between study groups at baseline were performed by the independent sample *t* test.

Due to the explorative study design, we performed no correction for multiple testing, and we considered a *p* value of <0.05 statistically significant. We used SPSS statistical software (SPSS), version 22, from Microsoft (SPSS, Inc., Chicago, USA) for statistical calculations and GraphPad Prism 5 (GraphPad Software, Inc., California, USA) for creating figures.

## Results

Initial statistical analysis showed no differences in mRNA transcripts between the WPC80 and the native whey groups in study 1, in neither young nor older participants. We therefore decided to combine the whey groups in further analysis. Furthermore, we combined the data sets from study 1 and study 2 because the design became similar in these two studies when merging the whey groups in study 1. Moreover, the mRNA responses were similar in the two studies. Thus, in this paper, we report the mRNA expression levels of selected genes in skeletal muscle and PBMCs from the two acute strength exercise studies combined where exercise was combined with supplementation with milk or whey protein.

Baseline characteristics in young and older subjects, independently of the supplement provided, showed that fat percent and leg lean mass were significantly different between young and older participants (Table 2).

**Table 2:** Baseline characteristics, independent on supplements provided

	Young	Older	*p* value^a^
	(*n* = 42)	(*n* = 37)	
Age (years)	27.0 (25.4–28.6)	73.9 (72.7–75.1)	<0.001
Body mass (kg)	75.0 (70.9–79.2)	74.3 (69.9–78.7)	0.82
Lean mass (kg)	54.2 (50.9–57.5)	49.8 (46.4–53.1)	0.06
BMI (kg/m^2^)	24.1 (23.1–25.2)	24.4 (24.4–25.6)	0.69
Fat percent (%)	24.9 (22.3–27.4)	29.3 (26.8–31.8)	0.02
Leg lean mass (kg)	18.9 (17.6–20.1)	17.1 (15.9–18.4)	0.05

Values are expressed as means (95% confidence interval for mean)

*n* number of participants, *BMI* body mass index

^a^Tested with independent sample *t* test

### Adherence to the exercise protocols

The average training volume in study 1 was 9050 ± 2197 and 5634 ± 2307 kg for the young and older subjects, respectively. The relative workload was the same for both groups (8 RM). All exercise sessions were performed as planned, but some subjects, in both groups, needed assistance with the last repetition and some seconds extended break before the last set.

The average training volume in study 2 was 11852 ± 3083 and 5982 ± 2458 kg for the young and older subjects, respectively. The relative workload was similar for both groups (10 RM). In study 2, three young and two older subjects did not go through with the shoulder press exercise due to shoulder pain. Except for this adjustment, all exercise sessions were performed as planned.

### Effects of exercise and protein supplementation on mRNA expression

**Fig. 3:**
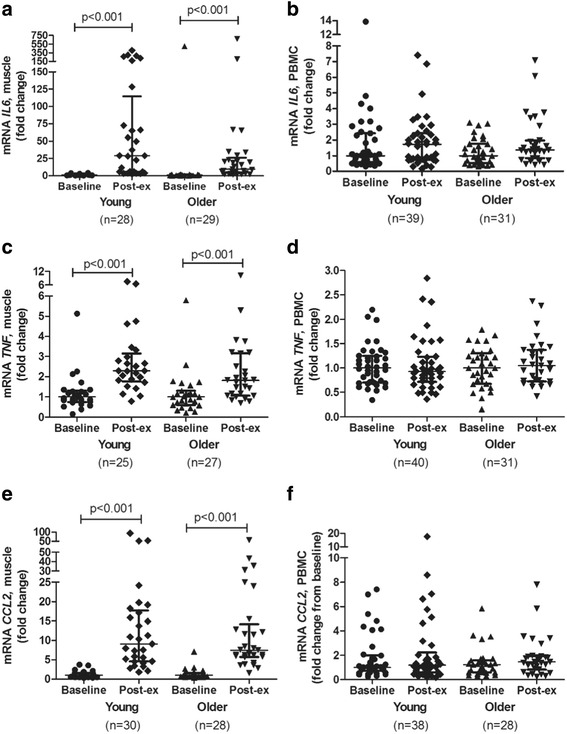
mRNA expression levels of *IL6* (**a**, **b**), *TNF* (**c**, **d**), and *CCL2* (**e**, **f**) after an acute bout of strength exercise, expressed as fold changes, in young and older subjects in skeletal muscle and PBMC. Skeletal muscle (young subjects); *n* = 25 (**c**), *n* = 28 (**a**), and *n* = 30 (**e**). Skeletal muscle (older subjects); *n* = 27 (**c**), *n* = 28 (**e**), and *n* = 29 (**a**). PBMC (young subjects); *n* = 40 (**d**, **f**) and *n* = 39 (**b**). PBMC (older subjects); *n* = 31 (**b**, **d**, **f**). Data are shown as median and interquartile ranges

**Fig. 4:**
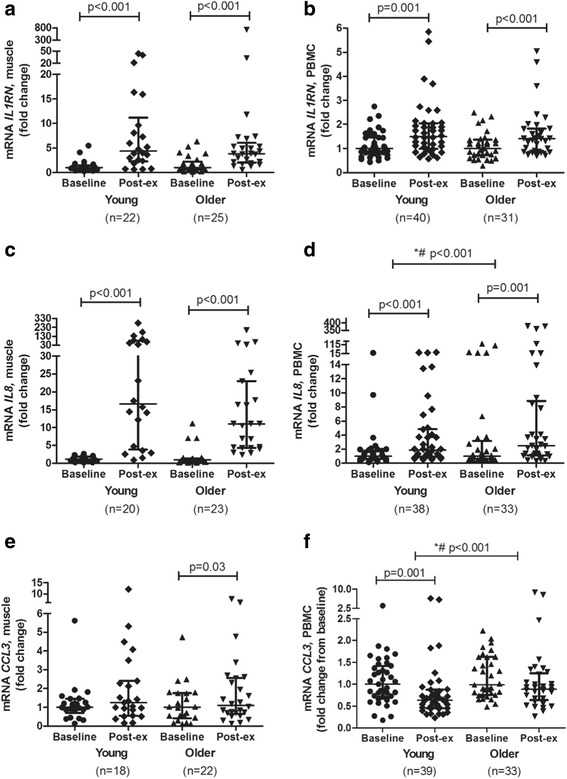
mRNA expression levels of *IL1RN* (**a**, **b**), *IL8* (**c**, **d**), and *CCL3* (**e**, **f**) after an acute bout of strength exercise, expressed as fold changes, in young and older subjects in skeletal muscle and PBMC. Skeletal muscle (young people); *n* = 18 (**e**), *n* = 20 (**c**), and *n* = 22 (**a**). Skeletal muscle (older subjects); *n* = 22 (**e**), *n* = 23 (**c**), *n* = 25 (**a**). PBMC (young subjects); *n* = 38 (**d**), *n* = 39 (**f**), and *n* = 40 (**b**). PBMC (older subjects); *n* = 31 (**b**) and *n* = 33 (**d**, **f**). Data are shown as median and interquartile ranges. *Indicates differences between young and older subjects. #Indicates differences at baseline between young and older participants

**Fig. 5:**
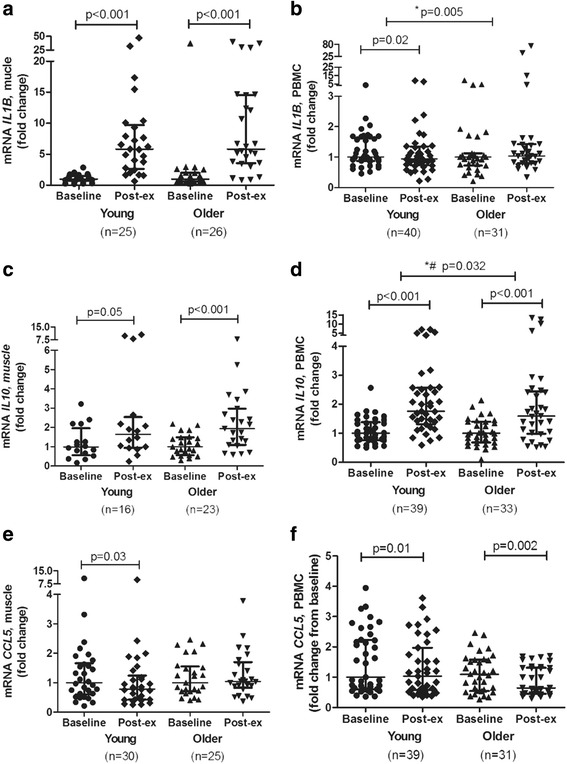
mRNA expression levels of *IL1β* (**a**, **b**), *IL10* (**c**, **d**), and *CCL5* (**e**, **f**) after an acute bout of strength exercise, expressed as fold changes, in young and older subjects in skeletal muscle and PBMC. Skeletal muscle (young people); *n* = 16 (**c**), *n* = 25 (**a**), and *n* = 30 (**e**). Skeletal muscle (older subjects); *n* = 23 (**c**), *n* = 25 (**e**), and *n* = 26 (**a**). PBMC (young subjects); *n* = 39 (**d**, **f**) and *n* = 40 (**b**). PBMC (older subjects); *n* = 31 (**b**, **f**) and *n* = 33 (**d**). Data are shown as median and interquartile ranges. *Indicates differences between young and older subjects. #Indicates differences at baseline between young and older participants

We observed that the mRNA expression levels of *IL6*, *TNF*, and *CCL2* in skeletal muscle increased significantly after acute exercise compared with smaller or no increase in mRNA expression levels in PBMCs (Fig. 3). The mRNA expression levels of *IL1RN*, *IL8*, and IL10 increased significantly in both skeletal muscle and PBMCs after exercise, but the expression levels of *IL1RN* and *IL8* were higher expressed in skeletal muscle after exercise than in PBMCs (Figs. 4a–d and 5c–d). The expression level of *IL10* was similar in skeletal muscle and PBMCs. The mRNA expression level of *IL1β* was significantly enhanced by exercise in skeletal muscle, whereas a significant increase of *IL1β* after exercise in PBMCs was observed in younger participants only (Fig. 5a–b).

Older participants showed an attenuated response in the mRNA expression level of *IL10* in PBMCs, whereas the mRNA expression levels of *IL8* and *IL1β* were higher in older compared to young participants. The mRNA expression level of *CCL3* decreased in both young and older participants, but the decrease was less pronounced in older participants. No significant changes in mRNA expression levels were observed between young and older participants in skeletal muscle (Figs. 4a, c, e, and 5a, c, e). Baseline mRNA expression levels of *IL8*, *IL10*, and *CCL3* in PBMCs were higher in older compared to younger participants. The mRNA expression levels in skeletal muscle and PBMCs of all genes measured, in both young and older subjects, are shown in the Additional file 2.

### Effect of exercise and protein supplementation on serum IL6

**Fig. 6:**
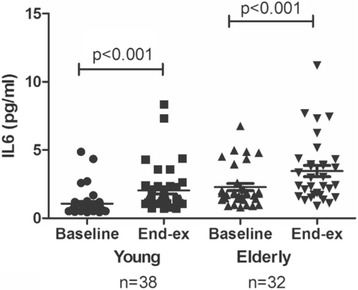
Serum levels of IL6 in young (*n* = 38) and older (*n* = 32) subjects at baseline and 1 h after exercise. Data are shown as median and interquartile ranges. n=number of participants

We observed a significant increase in the circulating level of IL6 after exercise in both young and older subjects (Fig. 6), with no significant difference between the two groups. Serum concentration of IL6 was significantly higher in older than in younger subjects at baseline (*p* < 0.001).

## Discussion

We observed that acute exercise modulated changes in mRNA expression levels of several genes known to be involved in repair, regeneration and adaptive processes of exercise, and in skeletal muscle as well as PBMCs. Some of the changes observed were regulated similarly in skeletal muscle and PBMCs, whereas other mRNA transcripts showed a unique pattern. Furthermore, we observed an attenuated response in the PBMC mRNA expression level of *IL10* in older compared to younger participants, whereas the PBMC mRNA expression levels of *IL8* and *IL1β* increased in older compared to younger participants after exercise. In contrast, the intake of different types of dairy protein had no significant impact on the mRNA response neither in skeletal muscle nor in PBMCs.

When comparing mRNA expression levels in skeletal muscle and PBMCs, we observed three different expression patterns: (i) mRNA transcripts increased significantly in skeletal muscle only, (ii) mRNA transcripts increased significantly in both skeletal muscle and PBMCs, but the magnitude of the increase was higher in skeletal muscle than in PBMCs, and (iii) mRNA transcripts were similarly expressed in both skeletal muscle and PBMC. These results demonstrate that skeletal muscle and PBMCs have overlapping, as well as unique mRNA responses to acute exercise, suggesting tissue-specific functions in response to acute exercise. IL6 has, for example, consistently been shown to increase in skeletal muscle and in serum after acute exercise [26, 45–49], whereas data from PBMC mRNA expression analysis showed a modest or no increase of IL6 after acute exercise [36]. A temporary increase in IL6 after an exercise may affect the satellite cells and promote myogenic lineage progression [50–53]. TNF and IL1β may have a role in promoting myoblast proliferation [54] and in inhibiting myoblast differentiation [55], potentially being important contributors to skeletal muscle adaptions. In PBMCs, the function of TNF and IL1β is primarily pro-inflammatory, playing a less important role after acute exercise. In addition, we observed a significant increase in mRNA expression levels of *IL10* and *IL1RN* after exercise in both skeletal muscle and PBMCs. We speculate that the increased mRNA expression levels of these cytokines may induce a regenerative response in patrolling PBMCs, possibly as an attempt to restore homeostasis [53, 56, 57]. The increase of these cytokines in PBMCs may be a result of IL6 released from skeletal muscle after acute exercise [57–60]. In the present study, the mRNA expression of *CCL2* was strongly increased in skeletal muscle, whereas no change was observed in PBMCs. Little is known about the function and physiological relevance of CCL2 after strength exercise in humans, but studies on contracting C2C12 myotubes show that CCL2 is released in a NF-κB-dependent manner to induce monocyte chemoattraction [61].

Few studies have been performed investigating the response to acute exercise in skeletal muscle and PBMCs simultaneously, but Liburt and colleagues observed that mRNA expression levels of *IL6* and *TNF* increased in skeletal muscle after acute exercise in horses, with no increase in PBMCs. They also found that the expression of *IL1* was similar in skeletal muscle and PBMCs [62]. Zeibig and colleagues found a significant correlation of mRNA transcripts of mitochondrial carnitine acyltransferases between skeletal muscle and human blood cells after 6 months of endurance exercise in young men [63], whereas Rudkowska and colleagues reported that 88% of the mRNA transcripts in skeletal muscle and PBMCs overlapped after 8 weeks of supplementation with n-3 polyunsaturated fatty acids using a transcriptome approach [9].

Further, we observed both a reduced and an increased response to acute exercise in older compared to younger participants in PBMCs, with no differences in skeletal muscle. Few, if any, have investigated possible differences in mRNA expression levels of PBMCs after acute exercise between young and older subjects. An attenuated cytokine response to acute exercise in older subjects has been observed in serum [33], with conflicting results in skeletal muscle [26, 28, 30, 64]. Knowing that the immune cells may be an important part of adaptive processes to exercise [30], an altered response of cytokines and chemokines in PBMCs of older subjects may ultimately impair regeneration. In the present study, the relative workload was the same in young and older participants, but the training volume differed between the groups. Younger participants were stronger and able to lift a higher load than the older participants. This may have contributed to a higher systemic stress in younger than in older participants, possibly being part of the explanation for the altered response observed in PBMCs between younger and older participants.

No differences in mRNA expression levels of investigated markers were observed 60–120 min after the heavy-load strength exercise, depending on the protein source (whey or milk). These results were supported by Nieman and colleagues who reported no differences in mRNA expression levels of markers, such as *IL6*, *IL1β*, *IL8*, and *IL10,* after an intense resistance exercise session, combined with the consumption of supplements consisting of carbohydrate (50%), protein (16%), and fat (34%) [12]. In contrast, other studies have indicated that whey proteins may have anti-inflammatory properties, by limiting the activation of NF-κB [21, 56]. In stimulated PBMCs, cultured in the presence of different glutamine concentrations, glutamine may enhance the production of T lymphocyte-derived cytokines, such as IL10 [15]. Similarly, glucose ingestion may attenuate IL6 release from contracting skeletal muscle after 120 min of cycling [16]. The time course of mRNA expression may differ in endurance and strength exercise, possibly explaining the different results.

There are some limitations to the present study. We report one post-exercise time point only, which limits our ability to identify potential differences in the time course of transcriptional regulation that may result from training or supplementation. This sampling point may also have been too early to detect possible differences in mRNA expression levels of the protein source provided. Another limitation of the study was that even though we are investigating the effect of a relative work load, because the young subjects were stronger and lifted approximately twice the volume of the old participants, it is not unreasonable to assume that the relative systemic stress (e.g., circulatory system) was higher in the young, and we therefore cannot exclude the possibility that this may have contributed to different responses in PBMC. Major strengths to the present study are the randomized controlled design, with participants receiving a standardized diet prior to, and on the test day, and the standardized exercise sessions that were performed under close supervision. The blood samples and muscle biopsies were collected simultaneously allowing comparison of the responses in the two tissues.

## Conclusions

We report changes in mRNA expression levels of selected genes, in skeletal muscle and PBMCs, after two acute bouts of strength exercise, followed by the intake of different protein supplements, in young and old participants. There were both overlapping and unique responses in mRNA transcripts of skeletal muscle and PBMCs in response to high-load exercise, suggesting tissue-specific functions in response to acute exercise. Furthermore, we observed that there were some differences in mRNA response to exercise in young and old subjects in PBMCs, possibly affecting regenerative mechanisms. Finally, our results show that different dairy protein supplements did not differentially alter mRNA transcripts after exercise.

## Additional files

Additional file 1: mRNA transcripts analyzed in both studies. Overview of mRNA transcripts analyzed in the present study. (DOCX 23 kb)
Additional file 2: mRNA expression levels in skeletal muscle and PBMCs of young and older subjects. Baseline and after exercise (post exercise) values, expressed as 2^−ΔCt.^. (DOCX 40 kb)

## Abbreviations

CCL: Chemokine (C-C motif) ligand
E%: Energy percent
IL: Interleukin
IL1RN: Interleukin 1 receptor antagonist
IMVC: Isometric maximum voluntary contraction
NF-κB: Nuclear factor kappa-light-chain-enhancer of activated B cells
NR4A2: Nuclear receptor subfamily 4, group A, member 2
PBMCs: Peripheral blood mononuclear cells
PPARGC1A: Peroxisome proliferator-activated receptor gamma coactivator 1-alpha
RM: Repetition maximum
RT-qPCR: Real-time quantitative polymerase chain reaction
TBP: TATA box-binding protein
TLDA: TaqMan Low-Density array
TLR2: Toll-like receptor 2
TNF: Tumor necrosis factor alpha
VO2max: Maximal oxygen uptake
WPC80: Whey protein concentrate
