# Supplementary material for: Gene expression is differentially regulated in skeletal muscle and circulating immune cells in response to an acute bout of high-load strength exercise
Source: Genes Nutr. 2017 Mar 3;12:8. doi: 10.1186/s12263-017-0556-4 (PMC5335818; doi:10.1186/s12263-017-0556-4)
Supplement: Additional file 1: — mRNA transcripts analyzed in both studies. Overview of mRNA transcripts analyzed in the present study. (DOCX 23 kb) [file 12263_2017_556_MOESM1_ESM.docx]

**Additional file 1**

Table S1 mRNA transcripts analyzed in both studies

| **Gene Symbol** | **Gene name** | **Entrez Gene ID (homo sapiens)** | **Assay ID** | **Function** |
| --- | --- | --- | --- | --- |
| ABCA1 | ATP-binding cassette; sub-family A, member 1 | 19 | Hs01059118_m1 | Transporter involved in the regulation of cholesterol, involved in inflammation |
| CCL2 | Chemokine (C-C-motif) ligand 2 | 6347 | Hs00234140_m1 | Involved in the acute response to exercise, important in chronic low-grade inflammation |
| CCL3 | Chemokine (C-C-motif) ligand 3 | 6348 | Hs00234142_m1 | Involved in the acute inflammation by recruitment and activation of leukocytes. |
| CCL5 | Chemokine (C-C-motif) ligand 5 | 6352 | Hs00982282_m1 | Involved in recruiting leukocytes to inflammatory sites. |
| CD36 | CD36 molecule | 948 | Hs01567185_m1 | Scavenger receptor involved in fatty acid metabolism |
| CXCL16 | Chemokine (C-X-C-Motif) Ligand 16 | 58191 | Hs00222859_m1 | Involved in the migration of cells, a chemoattractant |
| IL10 | Interleukin 10 | 3586 | Hs00961622_m1 | Down-regulates the expression of Th1 cytokines, enhances B cell survival, proliferation, and antibody production, able to block NF-κB activity |
| IL1*β* | Interleukin 1 beta | 3553 | Hs01555410_m1 | Proliferation and maturation of lymphocytes, involved in inflammation and acute-phase response |
| IL1RN | Interleukin 1 receptor antagonist | 3557 | Hs00893626_m1 | Inhibits the activities of IL1α/IL1*β*, and modulates a variety of interleukin 1 related immune and inflammatory responses |
| IL6 | Interleukin 6 | 3569 | Hs00985639_m1 | A pleiotropic cytokine that plays important roles in the acute-phase response of exercise and in chronic low-grade inflammation |
| IL8 | Interleukin 8 | 3576 | Hs00174103_m1 | Involved in angiogenesis in skeletal muscle, a chemoattractant |
| NR1H3 | Nuclear receptor subfamily 1; group H; member 3 | 10062 | Hs00172885_m1 | Transcription factor, involved in lipid metabolism and inflammation |
| NR4A2 | Nuclear receptor subfamily 4; group A; member 2 | 4929 | Hs00428691_m1 | Transcription factor, involved in energy metabolism and inflammation |
| NR4A3 | Nuclear receptor subfamily 4; group A; member 3 | 8013 | Hs00545009_g1 | Transcription factor, involved in energy metabolism and inflammation. |
| PPARGC1A | Peroxisome proliferator-activated receptor gamma; coactivator 1 alpha | 10891 | Hs01016719_m1 | Involved in energy metabolism and inflammation |
| TBP | TATA Box Binding Protein | 6908 | Hs00427620_m1 | General transcription factor that functions at the core of the DNA-binding multiprotein factor TFIID (used as housekeeping gene) |
| TLR2 | Toll-like receptor 2 | 7097 | Hs01872448_s1 | Involved in recognition of pathogen-associated molecular patterns, mediate the production of cytokines necessary for the development of effective immunity |
| TNF | Tumor Necrosis Factor | 7124 | Hs01113624_g1 | Prototypical pro-inflammatory cytokine, play a central role in inflammation, immune system development and apoptosis |
