# Supplementary material for: Gene expression is differentially regulated in skeletal muscle and circulating immune cells in response to an acute bout of high-load strength exercise
Source: Genes Nutr. 2017 Mar 3;12:8. doi: 10.1186/s12263-017-0556-4 (PMC5335818; doi:10.1186/s12263-017-0556-4)
Supplement: Additional file 2: — mRNA expression levels in skeletal muscle and PBMCs of young and older subjects. Baseline and after exercise (post exercise) values, expressed as 2−ΔCt.. (DOCX 40 kb) [file 12263_2017_556_MOESM2_ESM.docx]

**Additional file 2**

Table A2 mRNA expression levels in PBMCs of young subjects

| Gene | Timepoint | n  (20-40 yrs) | 2^-ΔCt^ values (percentiles) | | | p-values  (post exercise/ baseline) |
| --- | --- | --- | --- | --- | --- | --- |
|  |  |  | 25 | 50 | 75 |  |
| ABCA1 | Baseline | 39 | 0.215 | 0.294 | 0.401 | < 0.001 |
|  | Post exercise |  | 0.275 | 0.399 | 0.553 |  |
| CCL2 | Baseline | 38 | 0.037 | 0.046 | 0.089 | 0.55 |
|  | Post exercise |  | 0.029 | 0.052 | 0.096 |  |
| CCL3^*#^ | Baseline | 39 | 0.303 | 0.404 | 0.615 | 0.001 |
|  | Post exercise |  | 0.196 | 0.277 | 0.382 |  |
| CCL5 | Baseline | 39 | 80.90 | 135.49 | 301.91 | 0.01 |
|  | Post exercise |  | 79.15 | 135.08 | 259.32 |  |
| CD36 | Baseline | 41 | 7.46 | 9.24 | 12.20 | < 0.001 |
|  | Post exercise |  | 9.23 | 12.45 | 17.47 |  |
| CXCL16 | Baseline | 39 | 1.35 | 1.57 | 2.00 | < 0.001 |
|  | Post exercise |  | 1.77 | 2.25 | 3.12 |  |
| IL10 | Baseline | 39 | 0.010 | 0.013 | 0.019 | < 0.001 |
|  | Post exercise |  | 0.017 | 0.024 | 0.034 |  |
| IL1*β* | Baseline | 40 | 0.552 | 0.630 | 1.042 | 0.02 |
|  | Post exercise |  | 0.467 | 0.583 | 0.843 |  |
| IL1RN | Baseline | 40 | 1.47 | 1.89 | 2.74 | 0.001 |
|  | Post exercise |  | 1.81 | 2.83 | 3.88 |  |
| IL6 | Baseline | 39 | 0.005 | 0.008 | 0.019 | 0.11 |
|  | Post exercise |  | 0.006 | 0.013 | 0.019 |  |
| IL8^*#^ | Baseline | 38 | 0.022 | 0.034 | 0.061 | < 0.001 |
|  | Post exercise |  | 0.031 | 0.061 | 0.153 |  |
| NR1H3 | Baseline | 38 | 0.245 | 0.434 | 0.698 | 0.01 |
|  | Post exercise |  | 0.254 | 0.485 | 0.850 |  |
| NR4A2^#^ | Baseline | 40 | 0.030 | 0.041 | 0.060 | 0.47 |
|  | Post exercise |  | 0.028 | 0.037 | 0.052 |  |
| NR4A3 | Baseline | 40 | 0.014 | 0.023 | 0.036 | 0.01 |
|  | Post exercise |  | 0.014 | 0.026 | 0.045 |  |
| PPARGC1A^*^ | Baseline | 36 | 0.010 | 0.022 | 0.044 | 0.002 |
|  | Post exercise |  | 0.023 | 0.038 | 0.069 |  |
| TLR2 | Baseline | 41 | 3.10 | 4.64 | 5.56 | < 0.001 |
|  | Post exercise |  | 4.77 | 6.29 | 8.37 |  |
| TNF | Baseline | 40 | 1.07 | 1.52 | 1.90 | 0.80 |
|  | Post exercise |  | 1.10 | 1.42 | 1.92 |  |

n; number of participants. Values are calculated as 2^-ΔCt^, and shown at baseline and after exercise (post exercise). The mRNA expression levels were measured by quantitative real-time RT-PCR and normalized to the endogenous control TBP. p-values indicate changes between post exercise and baseline values. * indicate differences between young and older participants at baseline. # indicate differences post exercise between young and older participants.

Table A3 mRNA expression levels in PBMCs of older subjects

| Gene | Timepoint | n (≥ 70 yrs) | 2^-ΔCt^ values (percentiles) | | | p-values  (post exercise/ baseline) |
| --- | --- | --- | --- | --- | --- | --- |
|  |  |  | 25 | 50 | 75 |  |
| ABCA1 | Baseline | 30 | 0.198 | 0.306 | 0.394 | 0.02 |
|  | Post exercise |  | 0.243 | 0.323 | 0.596 |  |
| CCL2 | Baseline | 28 | 0.031 | 0.053 | 0.086 | 0.06 |
|  | Post exercise |  | 0.041 | 0.075 | 0.105 |  |
| CCL3^*#^ | Baseline | 33 | 0.442 | 0.590 | 0.960 | 0.18 |
|  | Post exercise |  | 0.375 | 0.521 | 0.737 |  |
| CCL5 | Baseline | 31 | 115.44 | 187.143 | 342.31 | 0.002 |
|  | Post exercise |  | 96.36 | 133.51 | 283.12 |  |
| CD36 | Baseline | 33 | 5.135 | 8.80 | 11.92 | < 0.001 |
|  | Post exercise |  | 9.48 | 13.03 | 18.26 |  |
| CXCL16 | Baseline | 33 | 1.39 | 1.87 | 2.50 | 0.001 |
|  | Post exercise |  | 1.80 | 2.47 | 3.60 |  |
| IL10 | Baseline | 33 | 0.011 | 0.017 | 0.024 | < 0.001 |
|  | Post exercise |  | 0.017 | 0.027 | 0.041 |  |
| IL1β | Baseline | 31 | 0.48 | 0.67 | 0.80 | 0.14 |
|  | Post exercise |  | 0.53 | 0.68 | 0.94 |  |
| IL1RN | Baseline | 31 | 1.38 | 1.89 | 2.59 | < 0.001 |
|  | Post exercise |  | 1.67 | 2.66 | 3.67 |  |
| IL6 | Baseline | 31 | 0.005 | 0.011 | 0.018 | 0.07 |
|  | Post exercise |  | 0.008 | 0.013 | 0.022 |  |
| IL8^*#^ | Baseline | 33 | 0.035 | 0.061 | 0.200 | 0.001 |
|  | Post exercise |  | 0.067 | 0.147 | 0.526 |  |
| NR1H3 | Baseline | 33 | 0.260 | 0.429 | 0.695 | 0.98 |
|  | Post exercise |  | 0.242 | 0.327 | 0.736 |  |
| NR4A2^#^ | Baseline | 34 | 0.032 | 0.047 | 0.070 | 0.07 |
|  | Post exercise |  | 0.034 | 0.058 | 0.086 |  |
| NR4A3 | Baseline | 34 | 0.018 | 0.023 | 0.033 | 0.46 |
|  | Post exercise |  | 0.015 | 0.026 | 0.044 |  |
| PPARGC1A^*^ | Baseline | 32 | 0.008 | 0.020 | 0.039 | 0.98 |
|  | Post exercise |  | 0.012 | 0.024 | 0.034 |  |
| TLR2 | Baseline | 34 | 2.87 | 4.12 | 5.24 | < 0.001 |
|  | Post exercise |  | 4.80 | 6.06 | 8.34 |  |
| TNF | Baseline | 31 | 1.16 | 1.54 | 2.06 | 0.34 |
|  | Post exercise |  | 1.11 | 1.61 | 2.11 |  |

n; number of participants. Values are calculated as 2^-ΔCt,^ and shown at baseline and after exercise (post exercise). The mRNA expression levels were measured by quantitative real-time RT-PCR and normalized to the endogenous control TBP. p-values indicate changes between post exercise and baseline values. * indicate differences between young and older participants at baseline. # indicate differences post exercise between young and older participants

Table A4 mRNA expression levels in skeletal muscle of young subjects

| Gene | Timepoint | n (20-40 yrs) | 2^-ΔCt^ values (percentiles) | | | p-values (post exercise/ baseline) |
| --- | --- | --- | --- | --- | --- | --- |
|  |  |  | 25 | 50 | 75 |  |
| ABCA1 | Baseline | 27 | 1.3 | 1.59 | 2.05 | 0.46 |
|  | Post exercise |  | 1.18 | 1.59 | 2.1 |  |
| CCL2 | Baseline | 30 | 0.23 | 0.34 | 0.55 | < 0.001 |
|  | Post exercise |  | 1.9 | 4.03 | 7.23 |  |
| CCL3 | Baseline | 18 | 0.006 | 0.014 | 0.021 | 0.27 |
|  | Post exercise |  | 0.008 | 0.018 | 0.034 |  |
| CCL5^#^ | Baseline | 30 | 0.138 | 0.236 | 0.424 | 0.03 |
|  | Post exercise |  | 0.113 | 0.206 | 0.318 |  |
| CD36 | Baseline | 28 | 24.76 | 40.07 | 44.09 | 0.04 |
|  | Post exercise |  | 25.65 | 32.66 | 57.7 |  |
| CXCL16^*^ | Baseline | 30 | 0.085 | 0.125 | 0.153 | < 0.001 |
|  | Post exercise |  | 0.128 | 0.204 | 0.368 |  |
| IL10 | Baseline | 16 | 0.004 | 0.005 | 0.008 | 0.05 |
|  | Post exercise |  | 0.005 | 0.008 | 0.012 |  |
| IL1β | Baseline | 25 | 0.007 | 0.012 | 0.017 | < 0.001 |
|  | Post exercise |  | 0.024 | 0.068 | 0.094 |  |
| IL1RN | Baseline | 22 | 0.007 | 0.013 | 0.018 | < 0.001 |
|  | Post exercise |  | 0.018 | 0.048 | 0.107 |  |
| IL6 | Baseline | 28 | 0.005 | 0.008 | 0.012 | < 0.001 |
|  | Post exercise |  | 0.046 | 0.238 | 1.055 |  |
| IL8 | Baseline | 20 | 0.006 | 0.01 | 0.016 | < 0.001 |
|  | Post exercise |  | 0.061 | 0.155 | 0.617 |  |
| NR1H3^*#^ | Baseline | 29 | 0.285 | 0.36 | 0.483 | 0.05 |
|  | Post exercise |  | 0.348 | 0.391 | 0.497 |  |
| NR4A2^*#^ | Baseline | 29 | 0.032 | 0.052 | 0.074 | < 0.001 |
|  | Post exercise |  | 4.772 | 8.00 | 22.46 |  |
| NR4A3^*^ | Baseline | 29 | 0.28 | 0.37 | 0.64 | < 0.001 |
|  | Post exercise |  | 73.1 | 108.38 | 121.72 |  |
| PPARGC1A^*^ | Baseline | 30 | 6.32 | 7.26 | 8.68 | < 0.001 |
|  | Post exercise |  | 10.4 | 12.82 | 41.7 |  |
| TLR2 | Baseline | 29 | 0.06 | 0.08 | 0.12 | 0.001 |
|  | Post exercise |  | 0.09 | 0.13 | 0.18 |  |
| TNF | Baseline | 25 | 0.02 | 0.03 | 0.04 | < 0.001 |
|  | Post exercise |  | 0.06 | 0.08 | 0.11 |  |

n; number of participants. Values are calculated as 2^-ΔCt^, and shown at baseline and after exercise (post exercise). The mRNA expression levels were measured by quantitative real-time RT-PCR and normalized to the endogenous control TBP. p-values indicate changes between post exercise and baseline values. * indicate differences between young and older participants at baseline. # indicate differences post exercise between young and older participants

Table A5 mRNA expression levels in skeletal muscle of older subjects

| Gene | Timepoint | n (≥ 70 yrs) | 2^-ΔCt^ values (percentiles) | | | p-values (post exercise/ baseline) |
| --- | --- | --- | --- | --- | --- | --- |
|  |  |  | 25 | 50 | 75 |  |
| ABCA1 | Baseline | 26 | 1.33 | 2.00 | 2.31 | 0.16 |
|  | Post exercise |  | 1.26 | 1.71 | 2.82 |  |
| CCL2 | Baseline | 28 | 0.25 | 0.38 | 0.57 | < 0.001 |
|  | Post exercise |  | 2.13 | 2.64 | 5.57 |  |
| CCL3 | Baseline | 22 | 0.006 | 0.014 | 0.025 | 0.03 |
|  | Post exercise |  | 0.01 | 0.014 | 0.035 |  |
| CCL5^#^ | Baseline | 25 | 0.175 | 0.264 | 0.454 | 0.66 |
|  | Post exercise |  | 0.265 | 0.316 | 0.536 |  |
| CD36 | Baseline | 27 | 29.87 | 41.49 | 61.89 | 0.02 |
|  | Post exercise |  | 31.63 | 46.75 | 75.11 |  |
| CXCL16^*^ | Baseline | 25 | 0.119 | 0.152 | 0.224 | 0.001 |
|  | Post exercise |  | 0.151 | 0.221 | 0.311 |  |
| IL10 | Baseline | 23 | 0.003 | 0.006 | 0.009 | < 0.001 |
|  | Post exercise |  | 0.006 | 0.01 | 0.016 |  |
| IL1β | Baseline | 26 | 0.005 | 0.008 | 0.017 | < 0.001 |
|  | Post exercise |  | 0.029 | 0.05 | 0.119 |  |
| IL1RN | Baseline | 25 | 0.006 | 0.011 | 0.032 | < 0.001 |
|  | Post exercise |  | 0.022 | 0.043 | 0.061 |  |
| IL6 | Baseline | 29 | 0.006 | 0.012 | 0.02 | < 0.001 |
|  | Post exercise |  | 0.041 | 0.124 | 0.332 |  |
| IL8 | Baseline | 23 | 0.004 | 0.01 | 0.015 | < 0.001 |
|  | Post exercise |  | 0.044 | 0.113 | 0.254 |  |
| NR1H3^*#^ | Baseline | 28 | 0.385 | 0.458 | 0.599 | 0.06 |
|  | Post exercise |  | 0.395 | 0.497 | 0.652 |  |
| NR4A2^*#^ | Baseline | 26 | 0.063 | 0.092 | 0.142 | < 0.001 |
|  | Post exercise |  | 1.14 | 2.442 | 9.845 |  |
| NR4A3^*^ | Baseline | 28 | 0.34 | 0.579 | 0.99 | < 0.001 |
|  | Post exercise |  | 44.10 | 98.98 | 122.49 |  |
| PPARGC1A^*^ | Baseline | 29 | 4.99 | 6.63 | 7.27 | < 0.001 |
|  | Post exercise |  | 10.34 | 21.48 | 58.79 |  |
| TLR2 | Baseline | 27 | 0.06 | 0.08 | 0.11 | 0.04 |
|  | Post exercise |  | 0.08 | 0.1 | 0.14 |  |
| TNF | Baseline | 27 | 0.02 | 0.04 | 0.06 | < 0.001 |
|  | Post exercise |  | 0.04 | 0.07 | 0.12 |  |

n; number of participants. Values are calculated as 2^-ΔCt^, and shown at baseline and after exercise (post exercise). The mRNA expression levels were measured by quantitative real-time RT-PCR and normalized to the endogenous control TBP. p-values indicate changes between post exercise and baseline values. * indicate differences between young and older participants at baseline. # indicate differences post exercise between young and older participants
